# Persistent cutaneous lesion in a child with tyrosinemia: Esomeprazole‐induced lichenoid drug eruptions

**DOI:** 10.1002/ccr3.4610

**Published:** 2021-08-16

**Authors:** Bahareh Abtahi‐Naeini, Hossein Saneian, Shakiba Dehghani

**Affiliations:** ^1^ Skin Diseases and Leishmaniasis Research Center Isfahan University of Medical Sciences Isfahan Iran; ^2^ Pediatric Dermatology Division of Department of Pediatrics Imam Hossein Children's Hospital Isfahan University of Medical Sciences Isfahan Iran; ^3^ Department of Pediatrics Imam Hossein Children’s Hospital Child Growth and Development Research Center Research Institute for Primordial Prevention of Non‐Communicable Disease Isfahan University of Medical Science Isfahan Iran; ^4^ School of Medicine Isfahan University of Medical Sciences Isfahan Iran

**Keywords:** child, esomeprazole, lichenoid drug eruption, proton‐pump inhibitors, tyrosinemia

## Abstract

Lichenoid drug eruptions (LDEs) are among well‐recognized adverse reactions that different drugs including Proton‐pump inhibitors (PPIs) are associated with. LDEs are rare adverse reaction of PPIs but they should be considered as appropriate management can lead to full resolution. Herein, we report a case of Esomeprazole‐induced LDE in a child with tyrosinemia.

## Graphical abstract

Lichenoid drug eruptions (LDEs) are cutaneous reactions to different drugs like proton‐pump inhibitors (PPIs). LDEs are rare adverse reaction of PPIs but they should be considered as appropriate management can lead to full resolution.

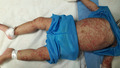

## 1 INTRODUCTION

Lichenoid drug eruptions are rare reaction to a medication, which are characterized by red to violaceous papules and plaques associated with pruritus. Lesion is typically symmetrical and has widespread distribution. Although LDEs are rare, they are among well‐documented cutaneous reactions. Most of the reports of LDEs are in adults, and presentation is uncommon in children. LDEs have been reported to be associated with some medications including Proton‐pump inhibitors (PPIs). ^1^

Proton‐pump inhibitors are one of the most widely prescribed GI drugs that are approved for the treatment of gastric acid‐related conditions. PPIs include omeprazole, esomeprazole, lansoprazole, rabeprazole, pantoprazole and dexlansoprazole. Although omeprazole and esomeprazole are different drugs, they share similarities in their clinical effects and subsequently adverse effects so there is a potential of cross reactivity between different components of family of PPIs.^2^

It is important that clinicians be aware of adverse cutaneous manifestations of esomeprazole to provide proper diagnosis and treatment.

We hereby describe a new case of LDEs in a 2‐year‐old girl with tyrosinemia treated with NEXIUM^®^ (esomeprazole magnesium).

## 2 CASE PRESENTATION

A 2‐year‐old girl was referred to pediatric dermatology clinic for evaluation of generalized skin lesions associated with severe pruritus from 4 weeks before that did not response to topical corticosteroid. Past medical history revealed that she was diagnosed with tyrosinemia and was under treatment by a pediatric Gastroenterologist. Due to the recent exacerbation of gastrointestinal reflux disorder (GERD), she was taking NEXIUM^®^ (esomeprazole magnesium) from 6 weeks ago. Her previous medications included nitisinone, vitamin K, vitamin E, vitamin A+D drop, iron drop, folic acid, and citric acid; potassium citrate oral solution, calcitriol, sodium phosphate, and zinc sulfate, which she had been taking for about 1.5 years. She had no history of allergies to medications.

**FIGURE 1:**
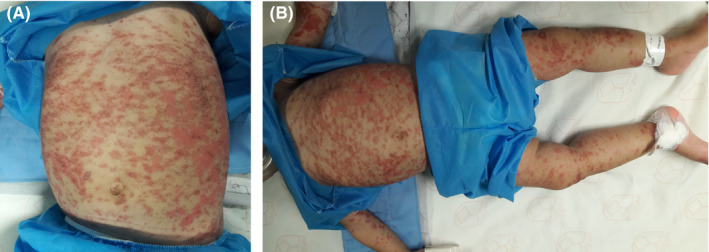
A, B, Lichenoid drug eruption. Widespread acute eczematous eruption on the trunk (A) and extremities (B)

**FIGURE 2:**
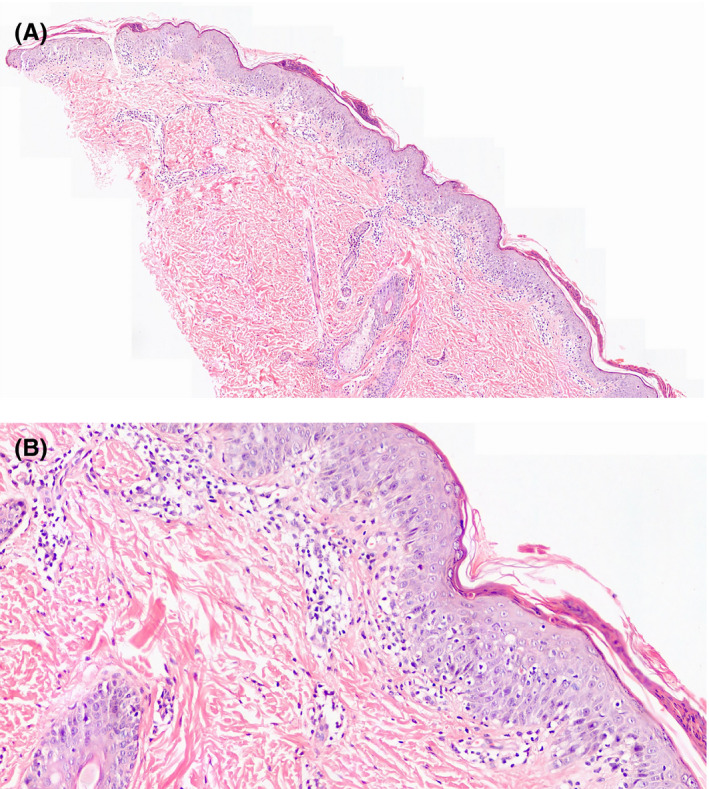
A, B, Histopathology of lichenoid drug eruption: Photomicrograph of skin biopsy of thigh demonstrates a lichenoid infiltration with basal layer vacuolation accompanied by some apoptotic cell (colloid bodies) in epidermis. (Hematoxylin and eosin)

Further questioning revealed no family history of lichen planus (LP), no history of hepatitis B vaccination, and no significant exposure to cinnamon‐containing products or foodstuffs. Her parents reported that the lesions were pruritic and she recently had sleep disturbance and irritability. On skin examination, widespread erythemato‐squamous plaques with eczematous features, associated with pruritus involving the trunk and extremities, were seen (Figure 1A, B). Buccal and genital mucosa were spare. There were no nail involvements. The clinical differential diagnosis included acute dermatitis, acute eczematous lichenoid eruption, and LP. A biopsy was performed on the left thigh and revealed parakeratosis, mild acanthosis, with spongiosis and basal layer vacuolation accompanied by some apoptotic cells in epidermis. In the dermis, there was an underlying lichenoid infiltration, which contained scattered eosinophil (Figure 2A,B). A histopathological diagnosis of LDE was made. Diagnosis of LDE related to the esomeprazole was probably suspected. A causality assessment was performed using the Naranjo Adverse Drug Reaction Probability Scale. (Table 1) Our patient's total Naranjo Scale score was 6 (possible adverse drug reaction). ^3^

**TABLE 1:** The Naranjo algorithm for adverse drug reaction for a patient with esomeprazole‐induced lichenoid drug eruption

Question	Yes	No	Do not know	Score
1. Are there previous conclusive reports on this reaction?	+1	0	0	0
2. Did the adverse event appear after the suspected drug was administered?	+2	−1	0	+2
3. Did the adverse event improve when the drug was discontinued or a specific antagonist was administered?	+1	0	0	+1
4. Did the adverse event reappear when the drug was readministered?	+2	−1	0	0 (do not know)
5. Are there alternative causes that could on their own have caused the reaction?	−1	+2	0	+2
6. Did the reaction reappear when a placebo was given?	−1	+1	0	0 (do not know)
7. Was the drug detected in blood or other fluids in concentrations known to be toxic?	+1	0	0	0 (do not know)
8. Was the reaction more severe when the dose was increased or less severe when the dose was decreased?	+1	0	0	0 (do not know)
9. Did the patient have a similar reaction to the same or similar drugs in any previous exposure?	+1	0	0	0 (do not know)
10. Was the adverse event confirmed by any objective evidence?	+1	0	0	+1
	Total Score: 6			

Note

Naranjo Adverse Drug Reaction Probability Scale: ≥9=definite adverse drug reaction; 5–8=probable adverse drug reaction; 1–4=possible adverse drug reaction; 0=doubtful adverse drug reaction.

Upon consultation with the patient's pediatric gastroenterologist, NEXIUM^®^ was discontinued. Oral prednisolone 1 mg/kg was initiated and continued for 3 weeks and then was gradually tapered. A topical steroid was also administered. Four weeks later, on follow‐up the lesions had improved significantly. The residual of brown hyperpigmentation on some areas persisted for over 1 year. A drug re‐challenge was not attempted due to her parents' refusal as to child's severe discomfort.

## 3 DISCUSSION

In the current paper, we reported a new pediatric case of Esomeprazole‐induced LDE in a 2‐year‐old girl with tyrosinemia that the diagnosis of LDEs was then approved by biopsy and histopathological results.

There are many known causative agents for LDEs including antimalarial, gold, nonsteroidal anti‐inflammatory agents, angiotensin‐converting enzyme inhibitors, thiazide diuretics, β‐blockers, and proton‐pump inhibitors.^4^

The clinical and histopathological manifestations of LDEs share similarities with those of lichen planus (LP), whereas they have differences regarding early lesion location, distribution, mucosal involvement, prognosis, and treatment. LDEs can be seen on skin, oral mucosa, or both. Oral lichenoid drug eruptions are extremely rare and challenging in diagnosis.^1^, ^5^

Proton‐pump inhibitors are among the most commonly prescribed drugs. They are widely and safely used but there are reports of skin reactions. Their common cutaneous adverse effects are immediate and delayed hypersensitivity reaction, Steven‐Johnson syndrome, toxic epidermal necrolysis, fixed drug eruption, vasculitis, and Sub‐acute cutaneous lupus erythematosus. Changes in Benzoimidazole ring on PPIs are believed to be the mechanism for hypersensitivity reactions associated with PPIs.^2^, ^6^

LDEs mostly affect elderly population and can have a latent period of weeks to a year depending on the previous exposure to the offending medication, its type and dosage. ^7^There are limited reports of PPI‐induced LDEs in literature. To our best of knowledge, none was a pediatric case, and none was induced by esomeprazole.

Bong. et al^8^ reported a case of PPI‐induced LDEs in a 81‐year‐old man who first developed annular scaly erythematous rash on his forearms, legs, and trunk 9 months after initiating treatment with omeprazole. He experienced the first recurrence 3 weeks after switching to lansoprazole and second recurrence several months after starting pantoprazole.

Brauer et al^7^ reported a similar case in a 78‐year‐old man whose initial eruptions were on his face, trunk, arms, and legs, and they had appeared shortly after medication change from lansoprazole to omeprazole.

In our patient, her underlying tyrosinemia, perhaps was a risk factor for cutaneous drug reactions and predisposed her to this generalized drug eruption and severe manifestations.

Although LDEs are a rare cutaneous reaction secondary to PPIs, it is important that recent lichenoid dermatitis in patients undergoing treatment with PPIs raise physicians' concern about the drug reaction, which can lead to providing proper diagnosis and treatment.

## CONFLICT OF INTEREST

None declared.

## AUTHOR CONTRIBUTIONS

Bahareh Abtahi‐Naeini had contributed in designing and conducting the study. Hossein Saneian had contributed in designing the study and manuscript revision. Shakiba Dehghani had assisted in preparation of the first draft of the manuscript. All authors have revised the manuscript critically for important intellectual content, also have read and approved the content of the manuscript and confirmed the accuracy or integrity of any part of the work.

## ETHICAL APPROVAL AND CONSENT TO PARTICIPATE

This report has been performed in accordance with the Declaration of Helsinki.

## CONSENT FOR PUBLICATION

Written informed consents were obtained from the patient for publication of this case report and any accompanying images.

## Data Availability

The datasets used during the current study are available from the corresponding author upon reasonable request.

## References

[1] Forouzan P, Riahi RR, Cohen PR. Atorvastatin‐induced lichenoid drug eruption: a case report and review of statin‐associated cutaneous adverse events. Cureus. 2020;12(3):e7155. 32257699 10.7759/cureus.7155 PMC7108677

[2] Salloum A, Nasr D, Maalouf D. Dermatologic adverse reactions to proton‐pump inhibitors: a synthetized review. J Cosmet Dermatol. 2021;20(4):1073‐1079. 33031621 10.1111/jocd.13763

[3] Naranjo CA, Busto U, Sellers EM, et al. A method for estimating the probability of adverse drug reactions. Clin Pharmacol Ther. 1981;30(2):239‐245. 7249508 10.1038/clpt.1981.154

[4] Ghosh SK. Generalized lichenoid drug eruption associated with imatinib mesylate therapy. Indian J Dermatol. 2013;58(5):388‐392. 24082187 10.4103/0019-5154.117315 PMC3778782

[5] Woo V, Bonks J, Borukhova L, Zegarelli D. Oral lichenoid drug eruption: a report of a pediatric case and review of the literature. Pediatr Dermatol. 2009;26(4):458‐464. 19689525 10.1111/j.1525-1470.2009.00953.x

[6] Yibirin M, De Oliveira D, Valera R, Plitt AE, Lutgen S. Adverse effects associated with proton pump inhibitor use. Cureus. 2021;13(1):e12759. 33614352 10.7759/cureus.12759 PMC7887997

[7] Brauer J, Votava HJ, Meehan S, Soter NA. Lichenoid drug eruption. Dermatol Online J. 2009;15(8):13. 19891921

[8] Bong JL, Lucke TW, Douglas WS. Lichenoid drug eruption with proton pump inhibitors. BMJ. 2000;320(7230):283. 10650025 10.1136/bmj.320.7230.283 PMC27275

